# Systematic review and meta-analysis comparing land and aquatic exercise for people with hip or knee arthritis on function, mobility and other health outcomes

**DOI:** 10.1186/1471-2474-12-123

**Published:** 2011-06-02

**Authors:** Stephanie I Batterham, Sophie Heywood, Jennifer L Keating

**Affiliations:** 1 Department of Physiotherapy, Monash University Peninsula campus, McMahons Rd, Frankston, Australia; 2 The Melbourne Sports Medicine Centre, 4/250 Collins St, Melbourne, Australia

## Abstract

**Background:**

Aquatic and land based exercise are frequently prescribed to maintain function for people with arthritis. The relative efficacy of these rehabilitation strategies for this population has not been established.

This review investigated the effects of aquatic compared to land based exercise on function, mobility or participants' perception of programs for people with arthritis.

**Methods:**

Medline, CINAHL, AMED and the Cochrane Central Register of Controlled Clinical Trials were searched up to July 2010. Ten randomised, controlled clinical trials that compared land to aquatic exercise for adults with arthritis were included. Study quality was assessed with the PEDro scale. Data relevant to the review question were systematically extracted by two independent reviewers. Standardised mean differences between groups for key outcomes were calculated. Meta-analyses were performed for function, mobility and indices that pooled health outcomes across multiple domains.

**Results:**

No differences in outcomes were observed for the two rehabilitation strategies in meta-analysis. There was considerable variability between trials in key program characteristics including prescribed exercises and design quality. Components of exercise programs were poorly reported by the majority of trials. No research was found that examined participant preferences for aquatic compared to land based exercise, identifying this as an area for further research.

**Conclusion:**

Outcomes following aquatic exercise for adults with arthritis appear comparable to land based exercise. When people are unable to exercise on land, or find land based exercise difficult, aquatic programs provide an enabling alternative strategy.

## Background

Aquatic therapy encompasses a range of approaches and may include passive immersion in mineral, hot or cold water. It may also incorporate the use of saunas, spas or exercise therapy. Aquatic exercise utilises the principles of hydrostatics and hydrodynamics to create challenges that promote health through exercise in water. The benefits of aquatic exercise are thought to result from water's unique characteristics including warmth that reduces pain and muscle spasm [1-3], buoyancy that decreases loading of joints [4], resistance to movement through turbulence and hydrostatic pressure, and the equal pressure from all directions applied to an immersed object at a given depth [3]. The unique characteristics of exercising in water may allow people to perform exercises that they would be unable to perform on land.

Arthritis is a process of inflammation and degeneration associated with pain, stiffness, joint instability and deformities that can significantly affect daily life. Arthritis is the major cause of disability and chronic pain in Australia with 3.85 million Australians affected every year. [5] It has no cure. Regular moderate physical activity provides a wide range of health benefits and graded exercise programs are effective interventions for knee osteoarthritis [6]. Both strength training and aerobic exercise lead to significant improvements in pain, physical function and general health although patient adherence to long term exercise is poor [7].

Geytenbeek (2002) found moderate quality evidence supporting aquatic exercise for pain, function, self efficacy, joint mobility, strength and balance outcomes for people with any disability [8]. The effects of aquatic exercise for people with arthritis were not investigated. In 2009, Bartels et al. reviewed the effectiveness of aquatic exercise for the treatment of knee and hip osteoarthritis compared to alternative strategies. The review included studies published till 2006 and concluded that aquatic exercise improves function (SMD = 0.26, 95% CI 0.11 to 0.42) and quality of life (SMD = 0.32, 95% CI 0.03 to 0.61) compared to no exercise control outcomes [9]. At that time only one study comparing aquatic to land exercise was available for review. Thomas et al. (2009) reviewed the treatment of knee osteoarthritis and concluded that aquatic exercise resulted in strength benefits compared to control interventions or land based exercise but did not quantify treatment effects [10]. Callahan (2009) evaluated all exercise interventions for participants with chronic arthritis and concluded that both aerobic and muscle strengthening exercises are safe and moderately effective for people with chronic arthritis [11]. Aquatic exercise appears to be a useful strategy for regaining movement and function loss associated with arthritis, but it is more expensive and resource intensive than land based exercise. It would be advantageous for those prescribing exercise to consider the nature and magnitude of effects on function when aquatic programs are compared to land based programs for this population. In addition, previous reviews have not summarized the content of exercise programs, making program replication difficult for readers.

### Objectives

This review explicitly assessed the relative advantages of aquatic exercise compared to land based exercise for people with arthritis on the outcomes of function or mobility. In addition this review sought data on participant perception of aquatic compared to land based exercise with respect to satisfaction, enjoyment and compliance. Data were extracted to identify how function and mobility outcomes of exercise programs have been measured and to summarise the components of reported land and aquatic exercise programs.

## Methods

### Inclusion Criteria

#### Types of Studies

Randomised controlled clinical trials were included. The study must have been reported in English as translation funding was not available. Studies must have reported that one group performed aquatic exercise and the comparison group participated in land based exercise; this could have included any exercise training for strength, endurance, resistance or aerobic capacity whether gym or home-based. To allow conclusions regarding the relative effects of aquatic and land exercise, papers were only included if they provided data that enabled outcomes following aquatic and land based exercise to be tested for significant differences.

#### Types of participants

Participants had to be people with rheumatoid arthritis or osteoarthritis.

#### Types of Outcome measures

Trials must have reported function, mobility or patient satisfaction outcomes using any assessment instruments.

### Exclusion Criteria

Trials in which participants performed aquatic or land based exercise in conjunction with other interventions were excluded unless the effects of aquatic compared to land based exercise could be partitioned from reported data. Participants less than 18 years of age were excluded due to the additional management implications associated with an immature musculoskeletal system. Participants who exercised as part of rehabilitation immediately following joint replacement surgery were excluded as the review focus was effectiveness for people with joints affected by arthritis.

### Search Strategy

Medline, CINAHL, AMED and the Cochrane Central Register of Controlled Clinical Trials were searched from the commencement of each database to July 2010. A sensitive search was developed using the terms 'aquatic physiotherapy', 'hydrotherapy' or 'water exercise' interventions for people with 'arthritis', 'osteoarthritis' or 'rheumatoid arthritis'. No terms relating to the 'comparison' and 'outcome' of trials were searched to avoid excessive exclusion of trials in an area where limited research has been conducted. The full electronic search strategy is available from the first author on request.

### Study Selection

Papers were initially screened and excluded based on title and abstract by two independent researchers. Full text was obtained for the remaining papers and these were assessed independently by both researchers against an inclusion and exclusion checklist. Disagreements were resolved through discussion; if this failed a third researcher was consulted.

### Quality Assessment

All included trials were critically appraised using the 11 item PEDro scale [12-14], 10 of which were scored using explicit decision rules. All trials were independently assessed by the first author. A search for included papers was then performed on PEDro and quality assessment scores compared to those determined by two independent PEDro assessors where these were available [15]. If there was disagreement on an item's assessment, these were assessed independently by another researcher. If no quality score was available in the PEDro database, the paper was independently assessed by both reviewers.

Item 4 (baseline comparability) was not fulfilled if there was a significant and important difference (95% confidence that SMD > 0.2) between groups at baseline for one measure of disease severity or one key outcome measure. If more than one outcome was measured by trials, only one outcome had to achieve baseline similarity to this fulfil criteria. Item 8 (key outcome measures were obtained for more than 85% of participants who were assessed at baseline) was calculated using data for each group (rather than for the pooled intervention and comparison group) when relevant data were reported.

### Data extraction

All data extraction and calculations were performed independently by two reviewers. Both sets of data were then compared for discrepancies and these were resolved through discussion.

The following data were systematically extracted: study design details, participant characteristics and baseline demographics, affected joints, duration of arthritis, group numbers, participant age and inclusion criteria, intervention and control group conditions including pool temperature, group size, supervision of the exercise intervention, provision of a home exercise program, compliance of participants, number of drop outs, length of interventions, duration and number of sessions, features and components of aquatic and land-based exercise including the provision of warm-up, stretching, cool down, balance, strengthening and functional exercises.

Data assessing function, pooled indices and mobility outcomes were also extracted. The World Health Organization defined six domains for the assessment of health [16]. These include pain, self care, usual activities, cognition, mobility and affect. Domains considered relevant to function were usual activities and self care. Outcomes that encompassed multiple domains were classified as pooled indices. Mobility was assessed through the extraction of data on walking ability and dynamic balance. If trials specified data collected under a range of walking speeds, data for the fast pace was extracted. To assess patient perception of the program any outcome that assessed patient enjoyment, satisfaction or any other type of feedback of the exercise programs was extracted.

To compare effectiveness of interventions for each relevant outcome, point measures and measures of variability were extracted. Means and standard deviations of outcomes measured immediately following the intervention were extracted and analysed. When necessary, the standard deviation [sd] was approximated by dividing the inter-quartile range by 1.35. Medians were used as best estimates of means. Standard error [SE] was converted to sd using the formula SE = sd/(√n). These data points were then used to calculate Hedges [17] corrected standardised mean differences [SMD] and 95% confidence intervals [CI] to assess intervention effects. The SMD was the difference between two means normalised using either pooled or control group standard deviations (the former where no significant difference in control and intervention standard deviations was observed). This index is useful for comparing data collected using different scales. A SMD <0.2 was considered a small effect, 0.5 (>0.2, <0.8) a moderate effect and >0.8 a large effect [18]. Data at baseline and immediately following the intervention were extracted. Long term effectiveness of interventions was not assessed as it was beyond the scope of the review. If trials did not use Intention-To-Treat analysis [ITT], per-protocol data were extracted for analysis.

### Meta analysis

Pooling of data across multiple studies can provide an improved estimate of the effect of the intervention as a consequence of the larger number of total participants and reduction in random error due to sampling differences.

Meta-analysis was performed using Review Manager (RevMan5) software[19]. Heterogeneity between trials was assessed using the I^2 ^statistic. Heterogeneity was considered substantial if I^2 ^was greater than 50% and a random effects model applied; otherwise a fixed effects model was used for the analysis [20]. SMDs were used where different scales were used to measure comparable outcomes across trials. Scale directions were aligned by adding negative values where required.

## Results

### Search yield

**Figure 1:**
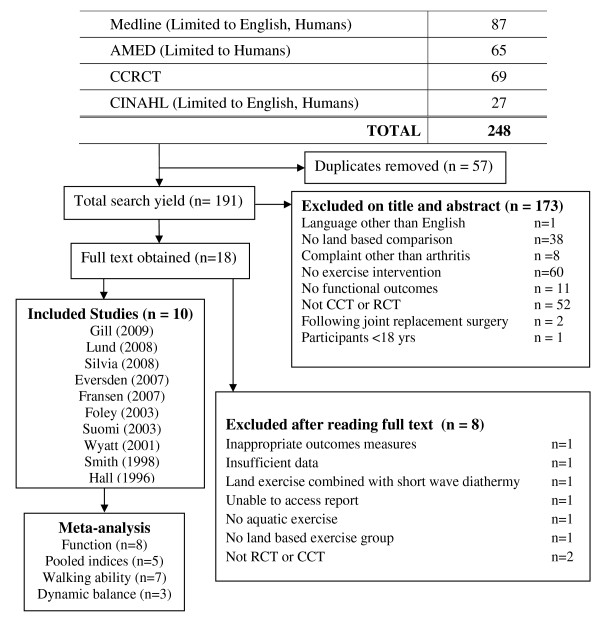
**Search yield**. CCT = Controlled Clinical Trial; RCT = Randomised Clinical Trial

A total of 191 papers were identified from the search. 173 papers were excluded based on title and abstract; full text was obtained for the remaining 18 papers. Of these 8 papers were excluded as they did not meet inclusion criteria and10 papers were included in the review. (Figure 1)

### Quality Assessment

A summary of the quality assessment scores and the decisions for each item are shown in Table 1 for all included trials.

**Table 1:** Summary of Quality assessment scores

	**Silva et al., 2008 **[2]	**Foley et al., 2003 **[1]	**Lund et al., 2008 **[27]	**Fransen et al., 2007 **[7]	**Wyatt et al., 2001 **[30]	**Suomi & Collier,2003 **[22]	**Eversden et al., 2007 **[23]	**Gill et al., 2009 **[25]	**Hall et al., 1996 **[21]	**Smith et al., 1998 **[31]
*1*. eligibility criteria	✓	✓	✓	✓	✓	✓	✓	✓	✓	✓

*2*. random allocation	**1 **drawing of lots	**1 **computer generated	**1 **opaque envelopes	**1 **computer generated	**1**	**1 **randomly assigned	**1 **flipping virtual coin	**1 **random numbers table	**1 **random numbers table	**1 **random allocation

*3*. allocation concealed	**1 **likely	**1 **sealed opaque envelopes,	**1 **baseline measures prior	**1 **after baseline assessment	**0 **not described	**0 **unlikely	**1 **sealed opaque envelopes	**1 **sealed envelopes	**1 **independent coordinator	**1 **independent allocator

*4*. baseline similarity	**1 **VAS (pain), WOMAC	**1 **Walk speed, ASE (pain)	**1 **VAS (pain), KOOS ADLs	**1 **WOMAC pain & function	**0**^**(1) **^no outcomes comparable	**1 **ADLs & Pain comparable	**1 **VAS (pain), EQ-SD	**1 **WOMAC pain & function	**1 **Knee ROM, AIMS2	**1 **Morning stiffness, HAQ

*5*. patient blinding	**0**	**0**	**0**	**0**	**0**	**0**	**0**	**0**	**0**	**0**

*6*. therapist blinding	**0**	**0**	**0**	**0**	**0**	**0**	**0**	**0**	**0**	**0**

*7*. assessor blinding	**1**	**1**	**1**	**1**	**1**	**0**	**1**	**1**	**1**	**1**

*8*. adequate follow-up	**0 **81% land	**0**^**(1) **^80% aquatic, 74% land	**0**^**(1) **^96% aquatic, 80% land	**1 **93% aquatic, 89% Tai chi	**1 **91% overall	**1 **91% patients each group	**0 **81% aquatic, 69% land	**1 **86% land 89% aquatic	**1 **94% overall	**0 **92% aquatic, 75% land

*9*. ITT analysis	**1**	**1**	**1**	**1**	**0**	**0**	**1**	**0**	**0**	**0**

*10*. between group comparisons reported	**1**	**1**	**1**	**1**	**1**	**1**	**1**	**1**	**0 **pre/post test	**1**

*11*. post intervention point & variability measures	**1 **means & SDs	**1 **medians & IQR	**1 **means & SDs	**1 **means & SDs	**1 **mean & SDs	**1 **means & SDs	**1 **medians & IQR	**1 **means & SDs	**1 **means & SDs	**1 **means & SDs

**TOTAL**	7/10*	**7/10**^(8)^	**7/10**^**(8)**^	8/10*	**5/10**^(6)^	5/10*	7/10*	7/10*	6/10*	6/10*

Key:

* accessed by PEDro with same score obtained

✓ yes (not scored)

^(x) ^PEDro assessment by PEDro reviewers differs and is x

**1 **yes (scored)

**0 **no

### Data Extraction

Study design features are shown in Table 2. Participant numbers reported in Table 2 are the numbers initially allocated to groups. Data describing the intervention design is shown in Table 3.

**Table 2:** Study design

		**Silva et al.,2008 **[2]	**Foley et al., 2003 **[1]	**Lund et al.,2008 **[27]	**Fransen et al., 2007 **[7]	**Wyatt et al.,2001 **[30]	**Suomi & Collier,2003 **[22]	**Eversden et al.,2007 **[23]	**Gill et al., 2009 **[25]	**Hall et al., 1996 **[21]	**Smith et al., 1998 **[31]
Intervention		PT	PT	PT	Tai chi, Hydro	PT	PACE AFAP	PT	PT + OT, education	PT	HEP (ROM), Hydro

Follow-up data reported				3 months	24 weeks			3 months	8 weeks	3 months	

Diagnosis		OA	OA	OA	OA	OA	RA & OA	RA	OA & RA	RA	RA

**Hip**		✗	✓	✗	✓	✗			✓		

**Knee**		✓	✓	✓	✓	✓			✓		

**Multiple joints**		✗	✗	✗	✗	✗			✗	>6	✓

Duration of arthritis years	**WB**			8.5 (3.7)*	≥1		23.5 (8.8)	10(7)*		9.7 (7.7)	20.2 (12.6)
			
	**LB**			7.8 (7.9)*			20.8 (7.7)	8(8.5)*		11.9 (8.2)	11.6 (7.6)

Number of subjects	**WB (n)**	32	35	27	55	46	11	57	44	35	12
			
	**LB (n)**	32	35	25	56		11	58	42	34	12
	
	**Control (n)**	✗	35	27	41	✗	10	✗	✗	Immersion 35 Relaxation 35	✗

Age	**WB**	59 (7.6)	73 (8.2)	65 (12.6)	70 (6.3)	45-70 yrs	68 (6.8)	55.2 (13.3)	69.2 (10.5)	55.8 (12.5)	61.9 (11.6)
			
	**LB**	59 (6.1)	69.8 (9.2)	68 (9.5)	70.8 (6.3)		64.2 (3.3)	56.1 (11.9)	71.6 (8.9)	58.5 (11)	54.9 (14.9)

Participant inclusion (✓) and exclusion (✗) criteria	Currently undertaking exercise	✗ if ≥3 sessions per 1/52 for >1/12	✗ if in exercise classes		✗ if >2 sessions per 1/52		✗ if classes in last 3/12		✓		✗
	
	Current PT	✗	✗					✗	✗	✗	
	
	Previous PT	✗ <6/12	✗ <6/52					✗ < 6/12	?	✓ > 30/7	
	
	Previous JRS		✗ < 12/12	✗	✗ <12/12		✗	✗ <3/12	✓	✗ <6/12	
	
	Awaiting JRS		✗ <12/52					✗	✓ 100%		
	
	Medications						stable	stable		stable	stable
	
	Corticosteroids	✗ <3/12			✗<3/12			✗ <4/52			
	
	Age (years)		>50		59-85	45-70	60-79	≥18			

*Key: *

✗ included

✓ participants (with this feature not included)

* mean (sd) estimated from median (IQR)

n number of participants

sd standard deviation

> more than

< within (designated time period)

/7 days

/12 months

/ 52 weeks

**AFAP **Arthistis Foundation Aquatic Program. **HEP **home exercise program. **JRS **joint replacement surgery. **LB **land-based intervention. **OA **osteoarthritis. **OT **occupational therapy home visit. **PACE **People with Arthritis Can Exercise. **PT **physiotherapy. **RA **rheumatoid arthritis. **ROM **range of movement. **WB **aquatic intervention.

All data presented as mean (sd) unless otherwise stated; missing data indicates that no relevant data were reported

**Table 3:** Intervention Design

	**Silva et al. 2008 **[2]		**Foley et al. 2003 **[1]		**Lund et al. 2008 **[27]		**Fransen et al. 2007 **[7]		**Wyatt et al.2001 **[30]		**Suomi & Collier 2003 **[22]		**Eversdenet al.2007 **[23]		**Gill et al. 2009 **[25]		**Hall et al. 1996 **[21]		**Smith et al. 1998 **[31]	
	
	WB	LB	WB	LB	WB	LB	WB	LB	WB	LB	WB	LB	WB	LB	WB	LB	WB	LB	WB	LB
***Exercise class size***	5-8						Max 15						1-4	1-6	4-6		4-5		<5	

***Supervision***	PT		✓	✓	PT students		PT	✓ Tai chi I	✓	✓	✓ AFAP I	✓ PACE I	✓	✓	PT	PT	PT	PT	PT	HEP

***Home program***	✗	✗	✗	✗	✗	✗	✗	✓video only	✗	✗	✗	✗	✓	✓	✓	✓	✗	✗	✗	✓

***Adverse effects %***			3	2	11	44		2							0	0				

***Drop-outs***	1	6	6	6	1	5	3	8	4		1	1	13	17	3	4	9		1	3

***Pool temperature ***°C	32	n/a		n/a	33.5	n/a	34	n/a	~32.2	n/a	31.7	n/a	35	n/a		n/a	36	n/a	36	n/a

***% Weight bearing/water depth***	1.2 m	n/a		n/a		n/a	~50%	n/a	~1.5 m	n/a	1.05 m	n/a		n/a		n/a		n/a	~20%	n/a

***Compliance%***	min. 80 required		84	75	92	85	81*	61*			79	90			82	88			89	

***Program time(weeks)***	18	18	6	6	8	8	12	12	6	6	8	8	6	6	6	6	4	4	10	10

***Session time (min)***	50	50	30	30	50	50	60	60			45	45	30	30	60	60	30	30	60	?

***Sessions per week***	3	3	3	3	2	2	2	2	3	3	2	2	1	1	2	2	2	2	3	x2-3/day

***Total number sessions***	54	54	18	18	16	16	24	24	18	18	16	16	6	6	12	12	8	8	30	140-210

*Key:*

✓ included

✗ not included in intervention

* % of participants who attended greater than 50% of classes

AFAP Arthritis Foundation Aquatic Program

HEP home exercise program (3 recheck appointments with physiotherapist)

I instructor

LB land based intervention

n/a not applicable

PACE People with Arthritis Can Exercise

PT Physiotherapist

WB aquatic intervention

Missing data indicates that no relevant data were reported

Features and components of aquatic and land based exercise interventions are described in Table 4. Hall et al. and Suomi & Collier did not provide any information about the exercise interventions and are not included in the table [21,22]. Suomi & Collier state that the programs were based on People with Arthritis Can Exercise (PACE) and Arthritis Foundation Aquatic Program (AFAP) protocols but these could not be found [22]. Hall et al. reported that exercises were designed to increase range of movement and muscle strength and that the type, duration and frequency of exercises were standardized in consultation with physiotherapists [21].

**Table 4:** Overview of exercise program components

		**Silva et al. 2008**[2]		**Foley et al. 2003**[1]		**Lund et al. 2008**[27]		**Fransen et al. 2007 **[7]		**Wyatt et al. 2001 **[30]		**Smith et al. 1998 **[31]		**Eversden et al. 2007**[23]		**Gill et al. 2009**[25]	
	
		WB	LB	WB	LB	WB	LB	WB	LB	WB	LB	WB	LB	WB	LB	WB	LB
***Warm-up***		✗	✗	✓ walking	✓ 4 min cycling	✓10 min running with belt	✓ 10 min cycling	✗	✓			✓ stretches		✓	✓		

***Stretches***		✓20s 2 reps	✓20s 2 reps	✓LL only	✓LL only	✓30s LL only	✓30s LL only	✗				✓	✓*	✓	✓	✓30s 2 sets LL	✓30s 2 sets LL

***Cool down***		✗	✗	✗	✗	✓5 min	✓5 min	✗				✓	✗	✓	✓	?	?

***Individually tailored exercises***		✗	✗	✓intensity only		✗	✗	✗				✓		✓individuals ability		✓	✓

***Reps/Sets strength exercises***		Isometric 7-10reps, 6s Isotonic 20-40reps		10reps ྡ3 × 15reps	10 RM ྡ 3 × 15reps	n of reps in 3.5 min for each exercise		10-20 reps		2 sets	2 sets		10 reps			✓	2 × 10reps

***Balance***		✗	✗	✗	✗	✓	✓trampoline, balance board, & cushion	✓		✗	✗	✗	✗			✗	✗

***Starting position specified***		✓	✓	✗	✗	✓	✓	✓	✗	✗	✗	✗	✗	✗	✗	✓	✓

***Aids***	***Weights***	✗	✓1 kg	✓	✓	✓	✓	✗								✗	✗
	
	***Elastic bands***	✗	✓			✗	✓	✗								✗	✗
	
	***Floats***	✓	n/a		n/a	✓	n/a	✓	n/a		n/a		n/a		n/a	✓	n/a
	
	***Turbulence***	✓	n/a	✓	n/a	✓	n/a	✓	n/a		n/a	✗	n/a		n/a	✓	n/a
	
	***Therapist***	✗	✗	✗	✗	✓	✗	✗	✗	✓	✓					✗	✗

Key:

✓ included

✗ not included

* 5 reps of 8-10 general ROM exercises

ྡ progressed to

LB land based intervention

LL lower limb

n/a not applicable

n number

reps repetitions

RM repetition maximum

WB aquatic intervention

There was considerable variability in the details provided about the exercise interventions. The majority of exercise programs were not detailed enough to allow them to be reproduced with confidence (Table 5).

**Table 5:** Variation in detail provided for exercise interventions across included studies

	**Silva et al.2008**[2]	**Foley et al.2003 **[1]	**Lund et al.2008 **[27]	**Fransen et al.2007 **[7]	**Wyatt et al.2001 **[30]	**Suomi & Collier2003 **[22]	**Eversden et al.2007 **[23]	**Gill et al.2009 **[25]	**Hall et al.1996 **[21]	**Smith et al.1998 **[31]
Aquatic exercise intervention	✓	✗	✓	✓	✗	✗✗	✗✗	✓	✗✗	✗

Land-based exercise intervention	✓	✗	✓	✗✗	✗	✗✗	✗✗	✓	✗✗	✗

Key:

✗ Some exercises reported

✗✗ No specific exercises reported

✓Adequately reported

Seven of the ten included trials presented information about exercises in their programs (Table 6). Eversden et al., Suomi and Collier and Hall et al. did not provide details of either program [21-23]. Eversden et al. stated that exercises focussed on joint mobility, muscle strength and functional activities [23]. Therefore none of these trials have been included in Table 6. Fransen et al. did not provide any details about the Tai Chi intervention [7].

**Table 6:** Exercise descriptions in included studies

		**Silva et al. 2008**[2]		**Foley et al. 2003**[1]		**Lund et al. 2008**[27]		**Fransen et al. 2007**[7]		**Wyatt et al. 2001**[30]		**Gill et al. 2009**[25]		**Smith et al. 1998 **[31]	
		
		WB	LB	WB	LB	WB	LB	WB	LB	WB	LB	WB	LB	WB	LB
***Aerobic Exercises***	***Running***					✓		✓	Tai chi					✓	
	
	***Jumping***					✓								✓	
	
	***Cycling***			✓	✓	✓	✓	✓				✓	✓20 min		
	
	***Walking mins***	✓10	✓10	✓				✓2		✓245 m	✓245 m	✓20-30	✓5-10		

***Exercises for strength***	***Hip flex.***	✓	✓	✓				✓				✓			
	
	***Hip ext.***	✓	✓	✓		✓	✓	✓				✓	✓		
	
	***Hip add.***	✓	✓	✓	✓	✓		✓				✓			
	
	***Hip abd.***	✓	✓✓	✓	✓	✓	✓	✓				✓			
	
	***Knee flex.***	✓	✓	✓		✓		✓		✓	✓	✓			
	
	***Knee ext.***	✓	✓	✓	✓	✓		✓		✓	✓				✓*
	
	***SBP***				✓										
	
	***DLP***				✓		✓								
	
	***Step-ups***						✓ 20 cm	✓ stairs					✓		
	
	***Sit/stand***						✓ 42 cm						✓		
	
	***SLR***	✓	✓							✓	✓				
	
	***Squats***							✓		✓	✓	✓			
	
	***Trunk flex.***		✓												
	
	***Dorsiflex.***	✓	✓												
	
	***Plantar flex.***	✓	✓												
	
	***Calf raises***		✓					✓				✓	✓		

Key:

✓ included

* isometric quads

abd. abduction

add. adduction

DLP double leg press

ext. extension

flex. flexion

LB land based intervention

m measured in meters not minutes

SBP seated bench press

SLR straight leg raise (hip flex., maintain end range knee extension)

WB aquatic intervention

### Intervention effects

#### Physical function

A number of outcomes were used to establish changes in participant's physical function (Additional File 1). The Western Ontario and McMaster Universities Osteoarthritis Index [WOMAC] has established reliability and validity for this population [24]. Function component scores as reported by Fransen et al., Gill et al. and Foley et al. were included [1,7,25]. Silva et al. reported a total WOMAC score; this result was included with pooled indices [2]. The Knee Injury and Osteoarthritis Outcome Score [KOOS] is based on the WOMAC [26,27]. The activities of daily life component score of the KOOS was used to assess function. The Health Assessment Questionnaire [HAQ] is a self reported disability measure related to activities of daily living [28]. The Arthritis Impact Measurement Scale 2 [AIMS2] assessed health status, and physical function subscale data was extracted [21].

#### Pooled indices

Indices that incorporated multiple domains of health were included as pooled indices (Additional File 2). Where multiple pooled indices were measured in a single trial, those measures that encompassed the greatest number of domains were pooled for meta-analysis. The outcomes that were included in the pooled indices assessment were the SF12, KOOS, Lequesne Index, WOMAC and EQ5D. The SF-12 is a subset of the SF-36 health survey and can be split into two components, a mental and physical component summary [29]. Wyatt et al. [30] did not measure a function or a pooled index and therefore was not included in *extracted data*.

#### Mobility

Walking ability was assessed by measuring walk speed (Additional File 3). All trials except Foley et al. reported time taken to walk a specific distance [1]. Foley et al. reported walk speed in ms^-1 ^without specifying distance walked [1]. The most common distance walked was between 10 m and 15 m however participants in some trials were assessed over 1609 m and 792 m. Dynamic balance incorporated functional aspects of mobility including the 30 second chair stand and the Timed Up & Go test. The Timed Up & Go test incorporates sit-to-stand, walking around a cone 3 m away and returning to sitting [7,22]. The 30 second chair stand involves timing the number of sit-to-stand repetitions possible in 30 seconds [25]. Lund et al. and Hall et al. did not report relevant outcomes, and were not included in *extracted data *[21,27].

#### Participants Perceptions

Hall et al. were the only authors to report measures of patient perception of the program [21]. No differences were observed in enjoyment of water compared to land based exercise programs.

### Meta analysis

**Figure 2:**
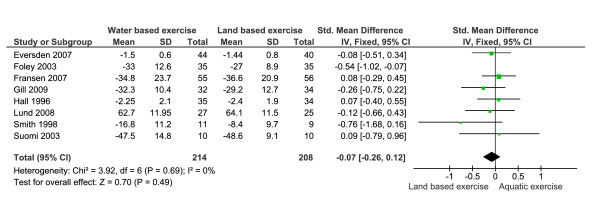
**Meta-analysis of function removing the data for Foley, et al.,**[1]**showing a non significant difference between the two exercise strategies**.

Meta-analysis was performed for function, mobility and pooled indices. Scale direction was adjusted as required using negative values; for function outcomes higher score indicated better health. For function outcomes a non significant SMD between groups of 0.13 (95% CI = -0.31, 0.04) in favour of land based exercise was found. As there were significant baseline differences between groups for Foley et al. SMD = 0.81 (95% CI = 0.33, 1.30) favouring land based exercise, meta-analysis was repeated excluding data from this study [1]. Removing this data improved the statistical heterogeneity (I^2 ^= 0%) of included trials (n = 249 aquatic, 243 land based) and resulted in an overall non-significant SMD of 0.07 (95% CI = -0.26, 0.12) (Figure 2).

**Figure 3:**
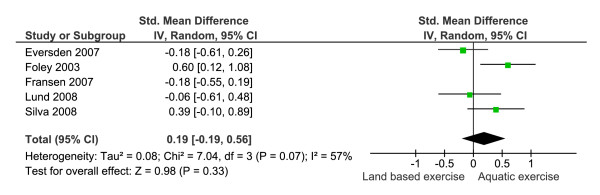
**Meta-analysis of outcomes that included multiple health domains removing the data for Fransen, et al. **[7]**showing a non significant difference between the two rehabilitation strategies**.

For indices that encompassed multiple health domains higher scores indicated better health. As significant statistical heterogeneity was detected (I^2 ^= 58%) a random effects model was used. No significant difference between groups was observed (SMD 0.10, 95% CI = -0.22, 0.42). A significant difference at baseline, SMD = -0.4 (95% CI = -0.03, 0.78) was detected for Fransen et al. As a result the analysis was repeated without this data [7]. Statistical heterogeneity was still significant (I^2 ^= 57%) and no significant difference between interventions (n = 136 aquatic, 132 land based) was detected (SMD 0.19 (95% CI = -0.19, 0.56) (Figure 3).

**Figure 4:**
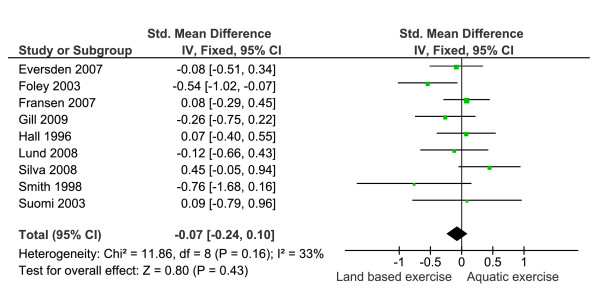
**Meta-analysis of function and indices which included multiple health domains showing a non significant effect for differences between the two exercise strategies**.

Function outcomes and indices that encompassed multiple domains of health were pooled (Figure 4). Nine trials (n = 281 aquatic and 275 land based) were included. Statistical heterogeneity was not significant (I^2 ^= 33%) and no significant difference between groups was observed (SMD 0.07 (95% CI = -0.24, 0.10)).

**Figure 5:**
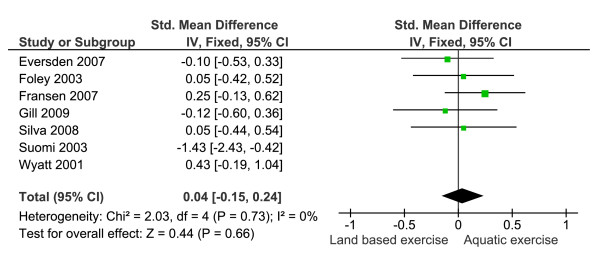
**Meta-analysis of walking ability without data for Suomi and Collier **[19]**and Wyatt, et al.**, [27]**showing a non significant difference between the two rehabilitation strategies**.

For mobility outcomes a smaller scores indicated better health. The pooled SMD was 0.03 (95% CI = -0.16, 0.21) indicating no statistically significant difference between exercise strategies. Suomi and Collier and Wyatt et al. both reported data that indicated significant between group differences at baseline [22,30]. When data were pooled without data from these trials heterogeneity improved (I^2 ^= 0%) but conclusions were unchanged (Figure 5). No significant difference between groups (n = 198 aquatic, 197 land based) was found (SMD = 0.04 (95% CI = -0.15, 0.24)).

**Figure 6:**
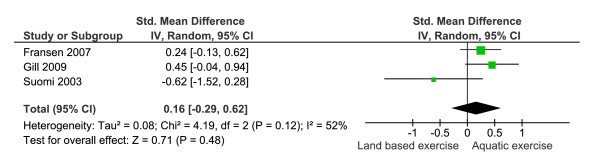
**Meta-analysis of dynamic balance showing a non significant difference between the two rehabilitation strategies**.

Three trials assessed dynamic balance or mobility [7,22,25] and all provided data that indicated baseline similarity between groups. Statistical heterogeneity was significant (I^2 ^= 52%) and a random effects model was applied (Figure 6). No significant difference between groups (n = 97 aquatic, 100 land based) was detected (SMD 0.16 (95% CI = -0.29, 0.62))

## Discussion

For all outcomes assessed in this review no statistically significant differences were found for outcomes following water based compared to land based exercise. This finding was not affected by exclusion or inclusion of trials with significant differences between groups prior to the intervention.

Although the majority of trials excluded participants with a history of surgery within the previous three months, some trials did not report against this characteristic. The results should therefore be considered to reflect outcomes for participants who were, in most cases, participating in treatment for arthritis.

Trials assessed a variety of measures relating to function, pooled indices and mobility. While a wide variety of functional outcomes were used, the WOMAC was the most common. The majority of trials assessed timed walks. Suomi and Collier and Wyatt et al. reported data for a 792 m and a 1609 m walk test respectively [22,30]. These longer distances require different training and may test walking endurance as well as speed. Both trials had significant group differences at baseline confounding a view of the effectiveness of interventions for this outcome.

Significant baseline differences distort post-intervention SMDs and may hinder a true reflection of between group differences. Significant baseline differences may also signal inadequate randomisation of participants. Analysis with and without trials with significant baseline differences did not change any review conclusions.

Only two trials, Foley et al. and Suomi and Collier found a statistically significant difference between land and aquatic exercise for any assessed outcome [1,22]. Foley et al. reported a significant difference between groups for function and pooled indices [1]. Significant baseline differences for function (SMD =-0.81, 95% CI -1.30, -0.33) favouring land based exercise) were found and attrition rates of 26% for land based and 20% of aquatic study participants further confounded a view of valid differences in intervention effects. Although Suomi and Collier found a significant intervention effect for the 792 m walk, assessment of the veracity of this result is confounded [22]. There were significant baseline differences and few participants (10 in each group).

### Exercise Intervention

Water and land both provide an environment for exercise. Regardless of the exercise medium, program goals can vary significantly. They may focus on improving function, range of movement or strength. Differences between land based programs are seen in descriptions of Tai Chi, gym or home based exercise programs. Aquatic programs also varied substantially (see Tables 4 &5).

Justification for the content of exercise interventions or exercise selection was rarely provided and few trials reported enough detail for both land and aquatic programs to be reproduced reliably.

No rationale was provided for the water depth at which exercises were performed and no authors reported the use of shallow water to progressively increase weight bearing and resistance. There was also no explanation for the inclusion or exclusion of balance and trunk control exercises. No authors reported the use of high intensity exercise in the water, for example with increased turbulence and speed. The resistance used in water exercise was also highly variable. Quantification of resistance applied in the aquatic programs through turbulence, buoyancy, therapists or elastic bands was not described in any of the trials. In the aquatic programs a wide variety of equipment was used to provide resistance including noodles, rings, kickboards, gaiters, balls, floats and aqua belts, but the reasons for these choices were not stated. Depth and temperature of water appeared similar across trials.

## Conclusion

Overall aquatic and land based exercises appeared to result in comparable outcomes for participants. Meta-analysis did not provide confidence that either aquatic or land based exercise provide better function or mobility outcomes. Variability in study parameters, study quality and exercise interventions may have contributed random error to outcomes, confounding the view of effects, however it is likely that both approaches yield comparable results. Most trials had design flaws, limiting confidence in observed effects. Three high quality trials (Silva et al., Smith et al. and Fransen et al.) each found no significant difference in outcomes for land compared to aquatic exercise [2,7,31].

High quality trial design, with intention-to-analysis, adequate follow-up and baseline similarity, would advance the quality of work in this field. Arguments for choice of exercise components and rationale for exercise choice and parameters would advance the science of exercise in water. There is a lack of information on patient satisfaction or adherence to exercise interventions despite the importance of patient engagement in exercise programs.

### Clinical Applications

Both aquatic and land based exercise programs appear to result in comparable outcomes for function, mobility or pooled indices. The prescription of aquatic exercise for arthritic conditions may not be warranted due to the cost and limited availability of aquatic programs. For the blanket prescription of aquatic exercise for people with arthritis, high quality trials showing clear benefits of aquatic programs compared to land based programs are required. On the other hand aquatic exercise appears neither more nor less effective than land based exercise. For people who have significant mobility or function limitations and are unable to exercise on land, aquatic exercise appears to be a legitimate alternative that may enable people to successfully participate in exercise. Clinical decision making regarding exercise choice should consider patients' specific requirements and disabilities, patients' preferences, therapist expertise and best available evidence as well as practical considerations such as availability and cost.

## Abbreviations

**ADL**: Activities of Daily Life; **AIMS2**: Arthritis Impact Measurement Scale 2; **ASE**: Arthritis Self-Efficacy score; **CI**: Confidence Interval; **HAQ**: Health Assessment Questionnaire; **IQR**: Inter-quartile range; **KOOS**: Knee Injury and Osteoarthritis Outcome Score; **MCS**: Mental component Summary Scale; **PCS**: Physical Component Summary Scale; **QoL**: Quality of Life; **ROM**: Range of Movement; **SMD**: Standardised Mean Difference; **VAS**: Visual Analogue Scale; **WOMAC**: Western Ontario and McMaster Universities Osteoarthritis Index; **50FWT**: 50 Foot Walk Test.

## Competing interests

There are no financial or non-financial competing interests for any authors. The systematic review was completed as partial fulfilment of the Bachelor of Physiotherapy with Honours degree (Monash University).

## Authors' contributions

All authors participated in the design of the review and read and approved the final manuscript. SH repeated the selection of studies for inclusion in the review, data extraction and analysis. JK supervised the overall development of the review, clarified statistical analysis methods, assisted in the interpretation of results and writing of the manuscript and provided a third independent assessment where necessary. SB developed inclusion/exclusion criteria, performed the searches on the databases, designed tables for data extraction, performed data extraction and meta-analysis. Both authors wrote the text of the review.

## Pre-publication history

The pre-publication history for this paper can be accessed here:

http://www.biomedcentral.com/1471-2474/12/123/prepub

## Supplementary Material

Additional File 1 **Effects of intervention on function**. Click here for file

Additional File 2 **Effects of intervention on outcomes that encompass multiply health domains**. Click here for file

Additional File 3 **Effects of interventions on mobility**. Click here for file
