# Supplementary material for: Systematic review and meta-analysis comparing land and aquatic exercise for people with hip or knee arthritis on function, mobility and other health outcomes
Source: BMC Musculoskelet Disord. 2011 Jun 2;12:123. doi: 10.1186/1471-2474-12-123 (PMC3141607; doi:10.1186/1471-2474-12-123)
Supplement: Additional File 1 — Effects of intervention on function. [file 1471-2474-12-123-S1.DOC]

### *Additional File 1* Effects of interventions on function

|  | **Gill, et al. 2009[25]** | | **Lund, et al. 2008[27 ]** | | | | **Eversden, et al. 2007[ 23]** | | **Fransen et al. 2007[7]** | | **Foley et al. 2003[1]** | | **Suomi et al. 2003[22]** | | **Smith et al. 1998[31]** | | **Hall, et al.**  **1996[21]** | | |
| --- | --- | --- | --- | --- | --- | --- | --- | --- | --- | --- | --- | --- | --- | --- | --- | --- | --- | --- | --- |
|  | **WB** | **LB** | **WB** | **LB** | | | **WB** | **LB** | **WB** | **LB** | **WB** | **LB** | **WB** | **LB** | **WB** | **LB** | **WB** | **LB** | |
| **Outcome measure** | WOMAC Function | | KOOS: ADLs | | | | HAQ | | WOMAC Function | | WOMAC Function | | ADL function | | HAQ | | AIMS2  Physical capacity | | |
| Scale | 0-68 | | 0-100 *** | | | | 0-3 | | 0-68 | | 0-68 | | 29-116 | | 0-24 | | 0-10 | | |
| n at *baseline* | 32 | 34 | 27 | | | 25 | 57 | 57 | 55 | 56 | 35 | 35 | 11 | 11 | 12 | 12 | 35 | | 34 |
| Mean at *baseline* | 36.0 | 36.9 | 44.7 | 40.6 | | | 1.4* | 1.5* | 46.3 | 47.2 | 34* | 28* | 57.4 | 53 | 19.2 | 12.1 | 2.3 | 2.7 | |
| SD at *baseline* | 10.3 | 12.9 | 18.1 | 13.6 | | | 0.7* | 0.6* | 20.4 | 20.6 | 8* | 6.5* | 18.5 | 13.7 | 16.2 | 10.7 | 1.9 | 2.1 | |
| SMD (95% CI) at *baseline* | +0.08  (-0.41, 0.56) | | +0.25  (-0.30, 0.80) | | | | +0.20  (-0.17, 0.56) | | +0.04  (-0.33, 0.42) | | -0.81  (-1.30, -0.33) ^ | | -0.26  (-1.14, 0.62) | | -0.5  (-1.31, 0.31) | | +0.20  (-0.28, 0.67) | | |
| n *after exercise* | 32 | 34 | 27 | | 25 | | 44 | 40 | 55 | 56 | 35 | 35 | 10 | 10 | 11 | 9 | 35 | | 34 |
| Mean *after exercise* | 32.3 | 29.2 | 62.7 | 64.1 | | | 1.5* | 1.4* | 34.8 | 36.6 | 33.0* | 27.0* | 47.5 | 48.6 | 16.8 | 8.4 | 2.3 | 2.4 | |
| SD *after exercise* | 10.4 | 12.7 | 12.0** | 11.5** | | | 0.4* | 0.5* | 23.7 | 20.9 | 8.5* | 6.0* | 14.8 | 9.1 | 11.2 | 9.7 | 2.1 | 1.9 | |
| SMD (95% CI) *after exercise* | -0.26  (-0.75, 0.22) | | -0.12  (-0.66, 0.43) | | | | -0.13  (-0.56, 0.30) | | +0.08  (-0.29, 0.45) | | -0.81  (-1.29, -0.32) ^ | | +0.09  (-0.79, 0.96) | | -0.76  (-1.67, 0.15) | | +0.07  (-0.40, 0.55) | | |

###

*Key*: * estimate of mean & sd based on median & IQR n number of participants

** SE values converted to sd negative SMD indicates in favour of land based exercise

***increased score equivalent to increased health positive SMD indicates in favour of aquatic exercise

^ significant difference between groups sd standard deviation

CI confidence interval SMD standardized mean difference

LB land based intervention WB aquatic intervention
