# Supplementary material for: Systematic review and meta-analysis comparing land and aquatic exercise for people with hip or knee arthritis on function, mobility and other health outcomes
Source: BMC Musculoskelet Disord. 2011 Jun 2;12:123. doi: 10.1186/1471-2474-12-123 (PMC3141607; doi:10.1186/1471-2474-12-123)
Supplement: Additional File 2 — Effects of intervention on outcomes that encompass multiply health domains. [file 1471-2474-12-123-S2.DOC]

##### Additional File 2 Effects of interventions on outcomes that encompass multiple health domains

|  | **Lund et al.**  **2008[27]** | | **Silva et al.**  **2008[2]** | | **Eversden et al. 2007[23]** | | **Fransen et al. 2007[7]** | | **Foley et al.**  **2003[1]** | |
| --- | --- | --- | --- | --- | --- | --- | --- | --- | --- | --- |
| Group | **WB** | **LB** | **WB** | **LB** | **WB** | **LB** | **WB** | **LB** | **WB** | **LB** |
| **Outcome 1** | KOOS: QoL | | Lequesne Index | | EQ-5D VAS: QoL | | SF-12 PCS | | SF-12 PCS | |
| Scale | 0-100 ******* | | 0-24 | | 0–100******* | | Norm scores *** mean(SD): 50(10) | | Norm scores *** mean(SD): 50 (10) | |
| n at *baseline* | 27 | 25 | 32 | 32 | 57 | 57 | 55 | 56 | 35 | 35 |
| Mean at *baseline* | 63.7 | 57 | 12.0 | 12.2 | 70* | 74* | 31.9 | 35.6 | 31.4* | 30.7* |
| sd at *baseline* | 11.8 | 12.4 | 3.82 | 3.78 | 17.5* | 13* | 8.5 | 9.6 | 4.0* | 5.6* |
| SMD (95% CI) at *baseline* | +0.55 (-0.01, 1.10) | | +0.07 (-0.42, 0.56) | | -0.26 (-0.63, 0.11) | | -0.4 (-0.78, -0.03) ^ | | +0.14 (-0.33, 0.61) | |
| n *after exercise* | 27 | 25 | 32 | 32 | 42 | 40 | 55 | 56 | 35 | 35 |
| Mean *after exercise* | 43.0 | 43.8 | 6.7 | 8.6 | 73* | 77* | 35.7 | 37.6 | 37.1* | 31.4* |
| sd *after exercise* | 12.5** | 12.5** | 4.21 | 5.48 | 15* | 15* | 9.8 | 11.2 | 6.4* | 6.4* |
| SMD (95% CI) *after exercise* | -0.06 (-0.61, 0.48) | | +0.39 (-0.1, 0.89) | | -0.26 (-0.70, 0.17) | | -0.18 (-0.55, 0.19) | | +0.89 (0.40, 1.38) ^ | |
| **Outcome 2** |  | | WOMAC: Pain, Stiffness, Function | | EQ-5D Index: health status valuation | |  | |  | |
| Scale |  | | 0-96 | | 0-1*** | |  | |  | |
| n at *baseline* |  |  | 32 | 32 | 57 | 58 |  |  |  |  |
| Mean at *baseline* |  |  | 32.9 | 34.9 | 0.7* | 0.7* |  |  |  |  |
| sd at *baseline* |  |  | 14.0 | 12.6 | 0.1* | 0.1* |  |  |  |  |
| SMD (95% CI) at *baseline* |  | | +0.15 (-0.34, 0.64) | | 0 (-0.37, 0.37) | |  | |  | |
| n *after exercise* |  |  | 32 | 32 | 44 | 40 |  |  |  |  |
| Mean *after exercise* |  |  | 15.6 | 22.7 | 0.7* | 0.7* |  |  |  |  |
| sd *after exercise* |  |  | 12.6 | 18.3 | 0.1* | 0.1* |  |  |  |  |
| SMD (95% CI) *after exercise* |  | | +0.45 (-0.05, 0.94) | | +0.1 (-0.33, 0.53) | |  | |  | |

*Key*: ***** Estimate of mean (SD) based on median (IQR) LB land based intervention

** SE values converted to sd n number of participants

*** increased score equivalent to increased health Outcome 1 used for meta-analysis

^ Significant difference between groups Outcome 2 additional outcome measure reported by some trials

- SMD indicates in favour of land based exercise sd standard deviation

+ SMD indicates in favour of aquatic exercise SMD standardized mean difference

CI confidence interval WB aquatic intervention
