# Supplementary material for: Systematic review and meta-analysis comparing land and aquatic exercise for people with hip or knee arthritis on function, mobility and other health outcomes
Source: BMC Musculoskelet Disord. 2011 Jun 2;12:123. doi: 10.1186/1471-2474-12-123 (PMC3141607; doi:10.1186/1471-2474-12-123)
Supplement: Additional File 3 — Effects of interventions on mobility. [file 1471-2474-12-123-S3.DOC]

##### Additional File 3 Effects of interventions on mobility

|  | **Silva et al.**  **2008[2]** | | **Foley et al.**  **2003[1]** | | | **Gill et al.**  **2009[25]** | | **Fransen et al. 2007[7]** | | **Wyatt et al. 2001[30]** | | **Suomi et al. 2003[22]** | | **Eversden et al. 2007[23]** | |
| --- | --- | --- | --- | --- | --- | --- | --- | --- | --- | --- | --- | --- | --- | --- | --- |
| Group | **WB** | **LB** | **WB** | | **LB** | **WB** | **LB** | **WB** | **LB** | **WB** | **LB** | **WB** | **LB** | **WB** | **LB** |
| **Outcome 1: Walking** | 50FWT: fast pace | | Walk speed | | | 50FWT | | 50FWT | | Timed 1mile walk | | 792m walk: fast pace | | 10m walk time | |
| Units | Time taken (s) | | Speed (m/s) *** | | | Time taken | | Time taken (s) | | Time taken (s) | | Time taken (s) | | Time taken (s) | |
| n at *baseline* | 32 | 32 | 35 | 35 | | 31 | 34 | 55 | 56 | 23 | 23 | 11 | 11 | 57 | 58 |
| Mean at *baseline* | 8.7 | 8.6 | 1.0* | 1.0* | | 15.8 | 14.4 | 11.2 | 11.3 | 1248 | 1314 | 576 | 492 | 10.9* | 10.2* |
| sd at *baseline* | 10.3 | 10.3 | 0.3* | 0.2* | | 6.3 | 4.9 | 2.3 | 2.3 | 78 | 132 | 77.4 | 79.2 | 2.5* | 2.2* |
| SMD (95% CI) at *baseline* | -0.01  (-0.50, 0.48) | | +0.09  (-0.38, 0.56) | | | –0.25  (-0.74, 0.24) | | +0.04  (-0.33, 0.42) | | +0.60  (0.01, 1.19) ^ | | -0.98  (-1.19, -0.06) ^ | | -0.3  (-0.67, 0.07) | |
| n *after exercise* | 32 | 32 | 35 | 35 | | 32 | 34 | 55 | 56 | 21 | 21 | 10 | 10 | 44 | 40 |
| Mean *after exercise* | 7.36 | 7.8 | 1.1** | 1.1** | | 13.6 | 13.1 | 10.3 | 11.0 | 1134 | 1182 | 563 | 480 | 9.1* | 8.8* |
| sd *after exercise* | 8.66 | 9.2 | 0.25** | 0.2** | | 4.6 | 3.5 | 2.2 | 3.3 | 84 | 132 | 7.2 | 30 | 1.8* | 2.2* |
| SMD (95% CI) *after exercise* | +0.05  (-0.44, 0.54) | | +0.01  (-0.46, 0.48) | | | -0.12  (-0.60, 0.36) | | +0.25  (-0.13, 0.62) | | +0.43  (-0.17, 1.03) | | –1.43  (-2.41, -0.45) ^ | | -0.15  (-0.58, 0.28) | |
| **Outcome 2: Dynamic balance** |  | |  | | | 30s Chair stand | | Timed Up & Go | |  | | Timed Up & Go (2 circuits) | |  | |
| Units |  | |  | | | Time taken | | Time taken (s) | |  | | Time taken (s) | |  | |
| n at *baseline* |  |  |  |  | | 31 | 34 | 55 | 56 |  |  | 11 | 11 |  |  |
| Mean at *baseline* |  |  |  |  | | 6.5 | 6.8 | 8.9 | 9.1 |  |  | 33 | 32.3 |  |  |
| sd at *baseline* |  |  |  |  | | 3.4 | 3.6 | 2 | 2.4 |  |  | 5.8 | 9 |  |  |
| SMD (95% CI) at *baseline* |  | |  | | | +0.08  (-0.40, 0.57) | | +0.09  (-0.28, 0.46) | |  | | -0.09  (-0.97, 0.79) | |  | |
| n *after exercise* |  |  |  |  | | 32 | 34 | 55 | 56 |  |  | 10 | 10 |  |  |
| Mean *after exercise* |  |  |  |  | | 7.8 | 9.7 | 8.2 | 8.8 |  |  | 31.2 | 28.3 |  |  |
| sd *after exercise* |  |  |  |  | | 4 | 4.3 | 1.7 | 3.0 |  |  | 5.3 | 3.5 |  |  |
| SMD (95% CI) *after exercise* |  | |  | | | +0.45  (-0.04, 0.94) | | +0.24  (-0.13, 0.62) | |  | | -0.62  (-1.52, 0.28) | |  | |

*Key* *Estimate of mean (sd) based on median (IQR) CI confidence interval

** Estimated using change scores from graph (median & IQR) LB land based intervention

*** Increased score equivalent to increased health n number of participants

^ significant difference between groups sd standard deviation

- SMD indicates in favour of land based exercise SMD standardized mean difference

+ SMD indicates in favour of aquatic exercise WB aquatic intervention
